# Abattoir-based estimates of mycobacterial infections in Cameroon

**DOI:** 10.1038/srep24320

**Published:** 2016-04-14

**Authors:** N. F. Egbe, A. Muwonge, L. Ndip, R. F. Kelly, M. Sander, V. Tanya, V. Ngu Ngwa, I. G. Handel, A. Novak, R. Ngandalo, S. Mazeri, K. L. Morgan, A. Asuquo, B. M. de C. Bronsvoort

**Affiliations:** 1 Microbiology and Parasitology Unit, Faculty of Allied Medical Sciences, University of Calabar, Calabar, Nigeria; 2 Tuberculosis Reference Laboratory Bamenda, P.O. Box 586 Bamenda, Cameroon; 3 The Roslin Institute, Royal (Dick) School of Veterinary Studies, University of Edinburgh, Easter Bush, Midlothian, EH25 9RG, UK; 4 Laboratory for Emerging Infectious Diseases, University of Buea, Buea, Cameroon; 5 Department of Biomedical Sciences, Faculty of Health Sciences, University of Buea, Buea, Cameroon; 6 Farm Animal Clinical Sciences, School of Veterinary Medicine, University of Glasgow, Glasgow, G61 1QH, UK; 7 Cameroon Academy of Sciences, P.O. Box 1457 Yaoundé, Cameroon; 8 School of Veterinary Medicine and Sciences, B.P. 454, University of Ngaoundere, Ngaoundere, Cameroon; 9 Laboratoire de Recherches Vétérinaires et Zootechniques de Farcha, N’Djaména, Chad; 10 Institute of Ageing and Chronic Disease, University of Liverpool, Leahurst Campus, Neston, Wirral, CH64 7TE, UK

## Abstract

Mycobacteria cause major diseases including human tuberculosis, bovine tuberculosis and Johne’s disease. In livestock, the dominant species is *M. bovis* causing bovine tuberculosis (bTB), a disease of global zoonotic importance. In this study, we estimated the prevalence of Mycobacteria in slaughter cattle in Cameroon. A total of 2,346 cattle were examined in a cross-sectional study at four abattoirs in Cameroon. Up to three lesions per animal were collected for further study and a retropharyngeal lymph node was collected from a random sample of non-lesioned animals. Samples were cultured on Lowenstein Jensen media and the BACTEC MGIT 960 system, and identified using the Hain^®^ Genotype kits. A total of 207/2,346 cattle were identified with bTB-like lesions, representing 4.0% (45/1,129), 11.3% (106/935), 23.8% (38/160) and 14.8% (18/122) of the cattle in the Bamenda, Ngaoundere, Garoua and Maroua abattoirs respectively. The minimum estimated prevalence of *M. bovis* was 2.8% (1.9–3.9), 7.7% (6.1–9.6), 21.3% (15.2–28.4) and 13.1% (7.7–20.4) in the four abattoirs respectively. One *M. tuberculosis* and three *M. bovis* strains were recovered from non-lesioned animals. The high prevalence of *M. bovis* is of public health concern and limits the potential control options in this setting without a viable vaccine as an alternative.

The genus *Mycobacterium* belongs to the phylum *Actinobacteria* and is a large genus including over 153 recognized species of bacteria^1^. The genus includes a number of highly pathogenic species responsible for major human and animal epidemics including human and animal tuberculosis and leprosy. These are broadly grouped into those that form tuberculous lesions in their hosts and those that do not. Tuberculous mycobacteria are grouped in what is known as the *Mycobacterium tuberculosis* complex (MTBC)^2^. This group includes among other species *Mycobacterium tuberculosis* and *M. africanum* the cause of human tuberculosis and *M. bovis* the cause of both bovine (bTB) and zoonotic (zTB) tuberculosis^3^. There are many non-tuberculous mycobacterial species including *M. leprae* (causing human leprosy), *M. avium* subspecies *paratuberculosis* (causing Johne’s disease) as well as other environmental mycobacteria that can be isolated from lymph nodes of apparently healthy carcasses such as *M. avium, M. fortuitum* and *M. gordonae*^4^.

*M. bovis* causes a chronic recrudescent disease which impacts production, and milk quality in cattle^5^ and can be transmitted from cattle to humans through air borne spread or consumption of infected milk or meat causing pulmonary or extra pulmonary zTB^6^. The control and eradication of bTB has had varying success in high-income countries. Wildlife reservoirs, such as badgers the United Kingdom^7^ and possums in New Zealand^8^ have become major reservoirs complicating control. However, in most middle and low income countries there is limited or no control in cattle and a reliance on meat inspection to limit the public health risks^3^,^9^,^10^.

The World Health Organization’s (WHO) “END-TB” goal is to eliminate all forms of human tuberculosis as a public health problem by 2035. However, the contribution of zoonotic tuberculosis (zTB) to human tuberculosis is poorly described, particularly in sub-Saharan Africa (SSA), where a combination of endemic bovine tuberculosis (bTB), evolving human-animal interfaces (e.g. expanding dairy production and increasing global animal based protein consumption), lack of appropriate diagnostic tools and practices and personal circumstances that hamper health-seeking behaviour present considerable challenges. As part of achieving this goal it is essential to understand the scale of the risk posed by bTB in low and middle income countries which carry the majority of the burden.

In Cameroon, zTB risk is managed by post mortem meat inspection of carcasses in government-controlled slaughterhouses. This approach usually detects only the more advanced stages of the infection. Pathological changes become visible only as the immune response tries to wall off the infection and visible lesions develop particularly in the lymph nodes that drain the head and respiratory system^11^. Previous studies in Cameroon have reported lesion prevalence estimates between 0.18–4.25%^12^ but the Northwest Region (NWR) of the country has generally been <1% although more recent data has suggested an increasing trend in the NWR^13^ since 2003. The same authors also reported that 51% of the inspected lesions had acid-fast bacilli consistent with mycobacterial infection.

However, to date, there have not been any attempts to get a comprehensive contemporary sample across the major cattle producing regions of Cameroon or to type all the species cultured to estimate the prevalence of the different mycobacterial species present and factors associated with bTB-like lesions and *M. bovis* infection in a Central or West African country. The key objectives of this study were to estimate mycobacterial species prevalences across different ecological settings over the four major cattle keeping administrative Regions of Cameroon and identify factors associated with an increased risk of having bTB-like lesions such as age or breed. Infected cattle were linked to their geographical origin as reported by butchers and their isolates typed to species level to give a higher resolution picture of the distribution of mycobacteria species in general and *M. bovis* in particular across Cameroon.

## Materials and Methods

### Study population

**Figure 1:**
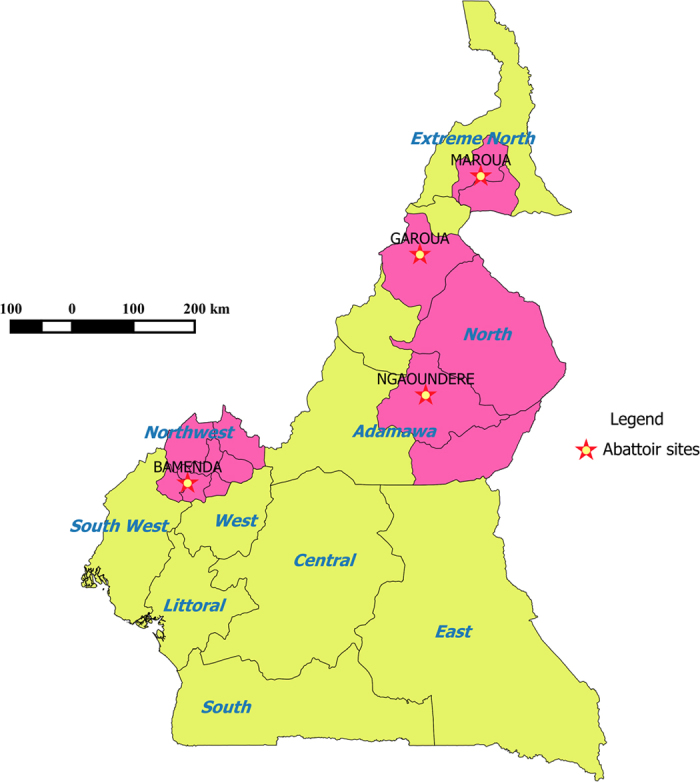
Map of Cameroon showing the 4 administrative Regions covered by the study and the location of the 4 municipal abattoirs where sampling took place (star). (Northwest Region, Adamawa Region, North Region and Extreme North Region). The pink areas are the lower administrative areas supplying cattle to these 4 abattoirs (*Generated using QGIS 2.2^®^ (*www.qgis.org*) and shp files obtained from the GADM database of Global Administrative Areas (*www.gadm.org)).

Cattle-keeping is an important livelihood for the Fulani ethnic group in the Northwest, Adamawa, North and Extreme North Regions of Cameroon (Fig. 1). There are no recent reliable figures for the cattle population of Cameroon but it is estimated to be ∼5–6 million head. The majority (∼42%) are in the high plateau areas of the Adamawa Region with a further ∼25% in the North and Extreme North Regions and remainder in the Northwest Region. The border between Cameroon, Chad, Central African Republic and Nigeria in the northern parts of Cameroon are extremely porous and many cattle come in from Chad, Sudan and The Central African Republic^14^,^15^,^16^,^17^, with many also leaving to Nigeria to feed its large human population. Cattle movements within Cameroon are not well documented but reflect the extensive nature of cattle management including seasonal transhumance^18^.

### Study design

The four municipal abattoirs of Bamenda (Northwest Region), Ngaoundere (Adamawa Region), Garoua (North Region) and Maroua (Extreme North Region) were selected for sampling as they were the largest abattoirs in each Region. It was anticipated that these were most likely to be receiving cattle predominantly from the local administrative area and so reflect largely what was happening in that Region in terms of circulating mycobacterial strains and thus allow some spatial resolution of strain diversity.

Based on previous estimates from the Northwest Region we assumed a prevalence of lesions of ∼5%^12^ and calculated a target sample size of ∼1,000 cattle per abattoir to ensure recovery of at least 25 isolates per abattoir assuming a 50% recovery from culture. This would allow the within abattoir prevalence of 5% to be estimated with a precision of ±1.3% with a 95% level of confidence. Due to project constraints, we were unable to sample in Garoua and Maroua abattoirs for more than a week each, which severely limited the final sample sizes from these Regions.

The working practices at the 4 abattoirs varied considerably and at each abattoir we spent time observing operations and piloting abattoir specific sampling protocols in order to minimize disruption to the work. However, the basic principles were the same: animals were cast for slaughter and restrained with ropes by the slaughter men; the research team immediately tagged it, collected a blood sample (results not presented here) and recorded animal-level data on owner/butcher, sex, age, dentition, body condition^19^ and origin. The slaughter men were given matching numbered tags for the offal so that at meat inspection the carcass, offal and head could be linked to the blood samples and animal data. The dentition score was based on the presence or absence of permanent incisor teeth with the scores ranging from 0 for no permanent incisor teeth present to 4 for all 4 pairs of incisors and an extra category, 5, for animals with broken teeth or excessively worn indicating older animals^20^.

Meat inspection was carried out following the 2002 MINEPIA meat inspection framework^21^. It should be noted that in Ngaoundere the research team did all the head inspections as these were not routinely being inspected for logistical reasons at this abattoir. Briefly, the lungs, liver and kidneys; lymph nodes of the thoracic and head regions; the mesenteric lymph nodes, other lymph nodes and tissues/organs of the body were visually examined, palpated and incised for the presence of granulomatous bTB-like lesions. Once identified by the veterinary inspectors, up to 3 macroscopic lesions from different anatomical sites per animal were collected into sterile 25 ml universal tubes using forceps and scalpel blades by our research team. Lesion type (purulent, caseous, or calcified), scale (single or multiple), size (<10 mm, 10–50 mm, >50 mm in diameter at widest axis) and pathological score (small lesion at 1 focus, small lesion at more than one focus, extensive necrosis) were also recorded following^22^,^23^. In addition to the tissue samples for culture from animals with lesions, a number of animals classed as non-lesioned by the meat inspectors at the Bamenda and Ngaoundere abattoirs were randomly sampled (using random number generator www.Random.org) and a single retropharyngeal lymph node per animal collected.

Tissue samples were stored on ice and transported back to the field lab or directly to the Tuberculosis Reference Laboratory (TBRL) in Bamenda. The samples from the Ngaoundere, Garoua and Maroua abattoirs were stored in liquid nitrogen and shipped to the TBRL Bamenda in dry shippers (Taylor-Wharton). Upon arrival at the TBRL, the samples were either thawed and processed immediately or stored at −80 °C until processed. The universal tubes containing samples were only re-opened at the TBRL in a Class II biological safety cabinet (BSC). Samples were processed in batches in the hood for efficiency (maximum of 8 samples per batch) but only a single sample opened at a time to avoid cross contamination.

### Mycobacterial culture

The tissue samples were prepared and cultured as described by the World Organisation of Animal Health (OIE)^24^ with minor modifications. Briefly, thawed samples were removed from their tubes in the BSC and fatty tissues were trimmed off using sterile forceps and scalpel blade and then the infected area (or just a random section of the lymph node for non-lesioned lymph nodes) of lymph node cut into tiny pieces. Three to five grams of the sliced tissue was then ground into a paste using a sterile mortar and pestle with the addition of 1–2 g of fine sterile sand and 5 ml of sterile normal saline (0.85%). The paste was then transferred to a 50 ml falcon tube and decontaminated by adding an equivalent volume of 4% NaOH and left to stand for 15 min with intermittent vortexing. Sterile PBS (pH 6.8, 0.067 M) was then added up to the 50 ml mark and the tube was centrifuged at 3200 g for 20 minutes at 18 °C. The supernatant was then decanted, and the resulting pellet was re-suspended in 2 ml of sterile PBS and ∼0.5 ml was inoculated on a Mycobacterial Growth Indicator Tubes (MGIT) using the BACTEC MGIT 960 automated culture system following manufacturer’s instructions, and ∼0.1 ml (2 drops) was also inoculated onto each of two slopes of Lowenstein Jensen (LJ) media (one supplemented with pyruvate and the other with glycerol). LJ cultures were observed for growth once a week until growth was observed, up to 12 weeks when it was classed as no growth observed.

The BACTEC MGIT 960 system includes a MGIT tube, MGIT 960 supplement kit and BACTEC MGIT 960 instrument. The MGIT tube contains 7 ml of modified Middlebrook 7H9 broth with casein peptone and an oxygen quenching fluorochrome (tris 4, 7-diphenyl-1, 10-phenonthroline ruthenium chloride pentahydrate) embedded in silicon at the bottom. The supplement kit contains antibiotics mixture -PANTA (Polymyxin B, Amphotericin B, Nalidixic acid, Trimethoprim, and Azlocillin) and an enrichment growth medium (OADC) containing oleic acid, bovine serum albumin with dextrose, catalase, and polyoxyethylene stearate. The MGIT instrument automatically monitors the fluorescence within each tube every hour to determine the culture status. A tube is indicated as positive when oxygen embedded in the fluorescent dye is used up by aerobic microorganism enabling the fluorochrome to fluoresce. The MGIT media was prepared according to the manufacturer’s instruction with the addition of 0.8 ml of PANTA/OADC mixture to each tube. Tubes were incubated for 8 weeks as recommended by the manufacturer^25^.

Any observed growth on the LJ medium or MGIT indicated positive growth were scraped and used to prepare a suspension in 3% formal saline, heat-fixed on a hot plate at 70 °C for 30 minutes, stained by the Ziehl-Neelsen (ZN) method^26^ and viewed under the 100× objective of a light microscope to determine the presence and morphology of acid-fast bacilli (AFB). MGIT Tubes that had no growth indicated by the BACTEC MGIT instrument after 56 days were visually examined for any growth before being discarded. If any growth was suspected a smear was also prepared, stained by ZN, and read to assess the presence of AFB.

### Typing of acid fast bacilli (AFB)

All AFB positive cultures were typed using the Hain GenoType MTBC assay and GenoType *Mycobacterium* CM/AS kits (Hain Lifescience, GmbH, Nehren, Germany). DNA was extracted from each positive culture using the GenoLyse kit (Hain Lifescience, GmbH, Nehren, Germany) strictly following the manufacturer instructions.

The Hain GenoType MTBC assay was performed following the manufacturer’s instructions and as previously described^27^. Briefly, 45 μl of a PCR mix comprising of 10 μl of amplification mix ‘A’ (containing biotinylated primers) and 35 μl of amplification mix ‘B’ was prepared in a 0.2 ml PCR tube. Five microliters of extracted DNA was added in a separate room to give a final reaction volume of 50 μl. Amplification was carried out in a thermal cycler (Applied Biosystem, 2720 Thermal cycler, USA) using the following protocol: denaturation at 95 °C for 15 min; 10 cycles of denaturation at 95 °C for 30 seconds and elongation at 58 °C for 120 seconds; an additional 20 cycles of denaturation at 95 °C for 25 seconds, annealing at 53 °C for 40 seconds, and elongation at 70 °C for 40 seconds; and a final extension at 70 °C for 8 min. Twenty microliters of the resulting amplification products were hybridized on labelled membrane strips at 45 °C for 30 min. Hybridized products were developed by addition of a conjugate buffer (containing streptavidin conjugated with alkaline phosphatase) and a subsequent addition of the substrate buffer for colorimetric detection of the bands. Species were identified according to their characteristic band pattern using the interpretation chart provided by the manufacturer (http://www.hain-lifescience.de/en/products/microbiology/mycobacteria/genotype-mtbc.html). Each MTBC strip contains 13 probes including amplification and hybridization controls to verify the test procedures. The kit with the exception of *M. tuberculosis*, *M. africanum* subtype II, and *M. canettii*, identifies unambiguously all of the MTBC species.

The GenoType *Mycobacterium* CM and AS assays detect 23 common NTM species frequently encountered in mycobacteriolosis plus an additional 14 species. This assay was done following the manufacturer’s instructions and as previously described^28^,^29^. Briefly, 45 μl of PCR mix comprising of 35 μl primer-nucleotide mixture (provided with the kit), 5 μl of 10× PCR Buffer, 2 μl of MgCl_2_, 0.2 μl of HotStarTaq polymerase (QIAGEN, Hilden, Germany) and 3 μl of molecular grade water was prepared in a 0.2 ml PCR tube. Five microliters of isolated DNA was added in a separate room to give a final reaction volume of 50 μl. The amplification protocol, hybridization and detection were same as for the GenoType MTBC assay described above. Identification of the non-tubercular mycobacteria (NTM) species were according to the interpretation table provided by the manufacturer (http://www.hain-lifescience.de/en/products/microbiology/mycobacteria/genotype-mycobacterium-cmas.html).

### Data management and statistical analysis

Data in the field and in the laboratory were recorded by hand and then transcribed to an Access database and spreadsheet respectively (Microsoft Corp.) and uploaded to Dropbox (www.dropbox.com). The R statistical software tool (www.R-project.org) was used to manipulate the data and for statistical analyses. Using R scripts the data was read and merged into new tables in a PostgresSQL (http://www.postgresql.org) database. Using R scripts extracts of data were made as csv files and passed to the R 3.2.0 (https://cran.r-project.org/) statistical environment for analysis.

Summary statistics were performed using the *epicalc* package. Prevalence and binomial 95% confidence intervals (exact method) were estimated using the *ci.binomial* function. Summary graphics were drawn using the *ggplot2* package. The chloropleth maps showing administrative Divisional/sub Divisional prevalences were produced using QGIS 2.2^®^ and shape files obtained from the GADM database of Global Administrative Areas (www.gadm.org). Principal component analysis (PCA) was carried out using the *princomp* function to collapse the 4 ordinal lesion scores (lesion type, scale, size and pathology score) into the first 2 unrotated components and the scores visualised stratified by abattoir using the *ggbiplot* function to explore for evidence of differences in pathology between abattoirs. Comparisons between abattoirs of the continuous PCA scores for the first 2 components were examined using ANOVA and pairwise comparisons were made using the *pairwise.t.test* function with a Bonferroni correction in the R statistical environment.

Univariable odds ratios for categorical variables for the binary outcome variable bTB-like lesion or not and *M. bovis* culture positive or not, were estimated using the *cci* function in the *epicalc* package. A new “age” variable was made by collapsing the dentition variable; juvenile = animal with 2 or less permanent incisors (dentition score of ≤2) and adult = an animal with three or more pairs of permanent incisor teeth (dentition score >2). A new condition score was also made: animals were categorised as ‘poor’ with a body condition score of less than 3, ‘normal’ for those with a condition score of 3 and ‘good’ for those with a score of 4 or 5.

An animal-level logistic regression model was developed to identify risk factors for the presence of bTB-like lesions and to test for differences in the prevalence between abattoirs after adjustment for other factors using the whole data set. The factors available were abattoir, breed, age, sex and condition score which were initially screened by univariable analysis. A second model was developed to identify risk factors for being *M. bovis* culture positive. Multivariable models were built by adding variables in a forward selection process in order starting with those with the smallest p-value from the univariable analysis. Variables with a p-value > 0.2 on the univariable analysis were not included in the multivariable analysis. Abattoir was forced into the model at the start to account for the stratification in the sampling design. The variables were allowed to be moved in and out to check if they were still significant (p < 0.05) after the addition of each new variable and their impact on the estimates checked (for potential confounding). The final model was compared to that based on the selection of the smallest Akaike Information Criterion (AIC)^30^ and if variables that were not significant based on p-value and Wald statistic improved the fit of the model, based on the AIC, they were included in the final model. Post model diagnostics were done using the *LogiDx* package.

### Ethical statement

This study did not involve the experimental use of any live vertebrate but reports the results of post mortem examinations of cattle for bovine tuberculosis carried out by the local veterinary inspectors in commercial abattoirs in Cameroon. Samples for culture were collected from carcases in accordance with best practice guidelines to minimise contamination. Local approval was given by the Head of Epidemiology at the Ministry of Livestock, Fisheries and Animal Industries responsible for supervision of activities at commercial slaughterhouses in each administrative Division. This project and the protocols had ethical approval from the University of Edinburgh Ethical Review Committee (Animal (Scientific Procedures) Act, 1986) (ERC No: OS02–13).

## Results

### Demographics of samples from the 4 municipal abattoirs in cameroon

**Figure 2:**
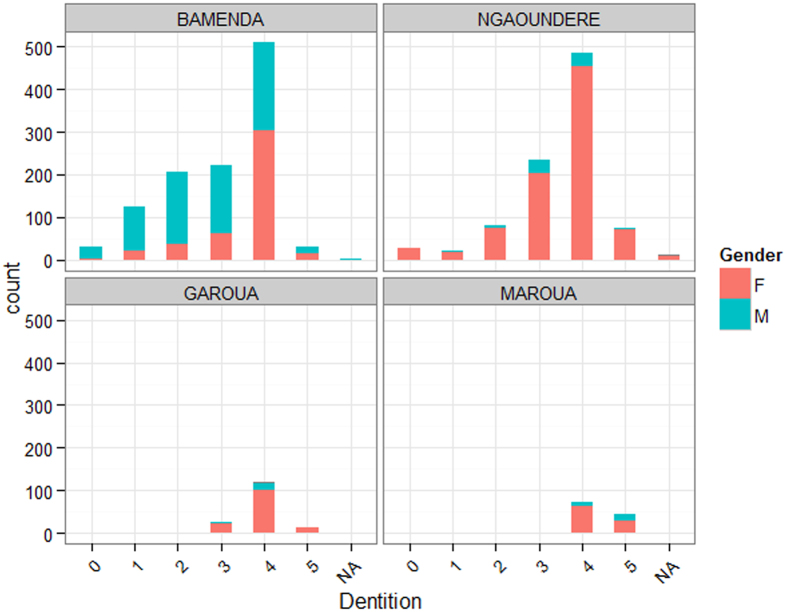
Age structure (based on dentition score) and sex of cattle slaughtered in the 4 municipal abattoirs in the study in Cameroon. (Animals with missing dentition data appear as grey band in Garoua). Totals for each abattoir were: Bamenda–1129; Ngaoundere–935; Garoua–160; and Maroua–122.

**Figure 3:**
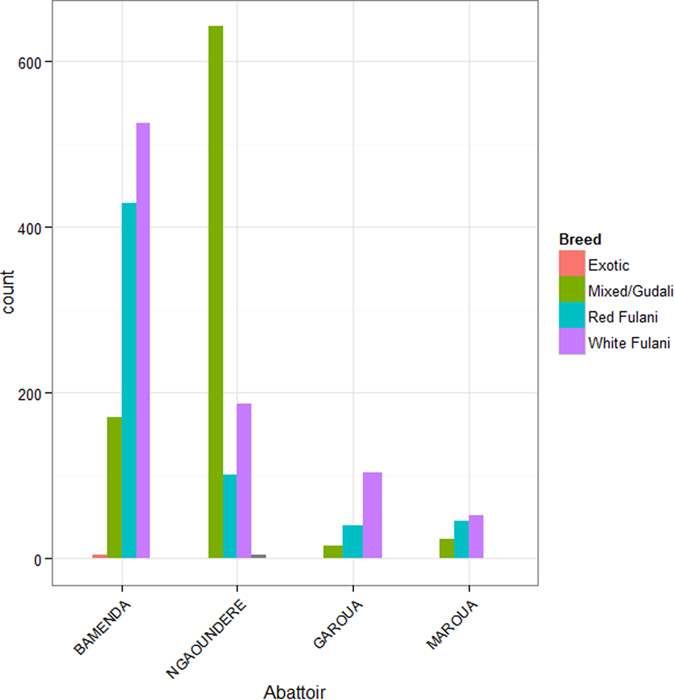
Histogram of the counts of the different breeds slaughtered at the 4 municipal abattoirs during the study periods in Cameroon. Totals for each abattoir were: Bamenda–1129; Ngaoundere–935; Garoua–160; and Maroua–122.

A total of 2,346 animals were examined from the four municipal abattoirs. This was divided across the abattoirs with 1,129 cattle sampled from Bamenda in a 3-month period in 2012 (April–June); 935 cattle from Ngaoundere in a 4 week period in August 2013; and 122 from Garoua and 160 from Maroua each sampled over just a week each in late September and October 2013 respectively. The summary statistics for age, breed and sex of the animals sampled by abattoir are given in Table 1 and the distribution of animal ages are plotted in Fig. 2. This data suggests there is a very marked difference between the Bamenda and the northern abattoirs. Bamenda appears to slaughter a much higher proportion of younger males in comparison to the northern abattoirs. In addition, there is a strong breed difference between the abattoirs with more Red and White Fulani cattle being slaughtered in the Northwest and more Gudali and mixed breeds in the northern abattoirs (Fig. 3).

### Prevalence of bTB-like lesions in slaughter cattle from the 4 municipal abattoirs in cameroon

**Figure 4:**
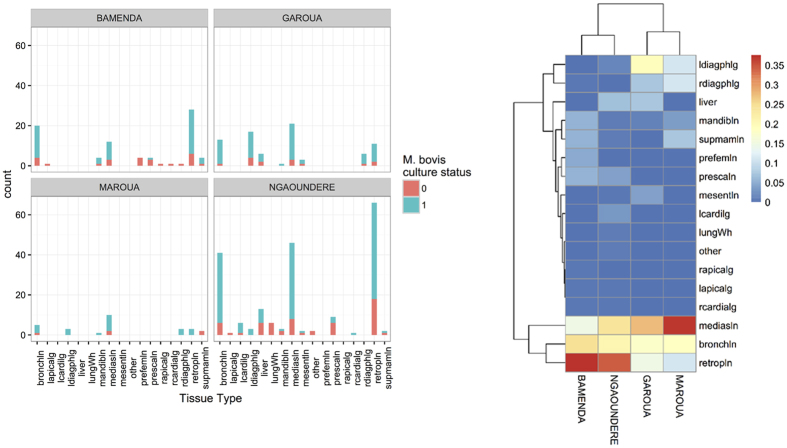
(**a**) Distribution of the raw counts of lesions found in the 207 cattle with one or more bTB-like lesion and whether lesion was culture positive for *M. bovis*, (**b**) Proportion of observed bTB-like lesions per site for the 207 cattle with bTB-like lesions stratified by abattoir to show different patterns in each of the 4 municipal abattoirs studied in Cameroon. The dendrogram branch lengths on the two axes reflected the relatedness of the abattoirs (x-axis) and lymph nodes (y-asis) in relation to distribution of *M. bovis*. Key: Mediasln = mediastinal LN; retropln = retropharyngeal LN; bronchln = bronchial LN; supmamln = supramammary LN; prefemln = prefemoral LN; prescaln = prescapular LN; mandibln = submandibular LN; rcardialg = right cardiac lung lobe; rdiagphlg = right diapgragmatic lung lobe; rapicalg = right apical lung lobe; lapicalg = left apical lung lobe; lungWh = whole lung affected; liver = liver lesions; mesentln = mesenteric LN; lcardilg = left cardiac lung lobe; ldiagphlg = left diaphragmatic lung lobe.

**Figure 5:**
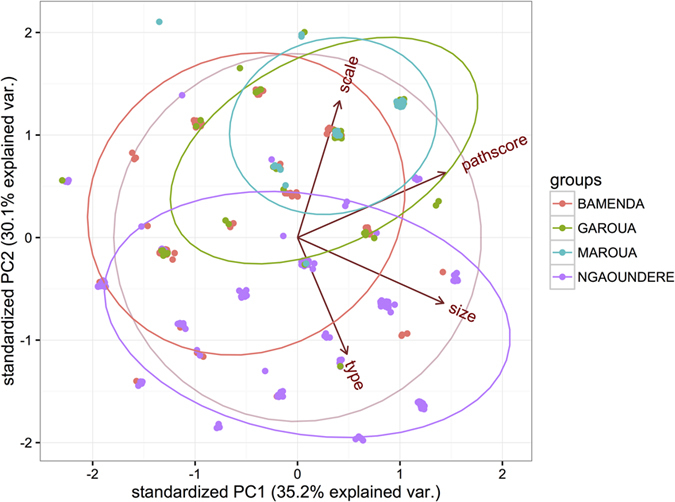
Plot of the first two principal components (PC1 and PC2) from analysis of the four lesion descriptive variables; lesion type (purulent, caseous, or calcified), scale (single or multiple), size (<10 mm, 10–50 mm, >50 mm in diameter at widest axis) and pathology score (=pathscore) (small lesion at 1 focus, small lesion at more than one focus, extensive necrosis). The points are jitter to reveal overlapping points and coloured by abattoir with 80% ellipses for each.

A total of 316 bTB-like lesions (mainly from lymph nodes) were collected from 207 of the 2,346 inspected animals. Due to logistical problems lesions from 6 animals in Ngaoundere did not get cultured so the denominator for cultures is 201. At least one tissue sample was collected from every animal that presented with one or more bTB-like lesions at different anatomic sites of the carcass. In addition, retropharyngeal lymph node samples were collected from a random sample of 179 apparently healthy animals with no visible lesions in the Bamenda and Ngaoundere abattoirs. Overall, bTB-like lesions were mostly observed in retropharyngeal, mediastinal and bronchial lymph nodes in all abattoirs (Fig. 4a). However, when the relative proportions are compared (Fig. 4b) there is a clear difference between abattoirs with a much higher proportion of lesions being in the retropharyngeal lymph nodes in Bamenda (0.376) and Ngaoundere (0.343) while mediastinal lesions predominate in Garoua (0.277) and Maroua (0.370). Also, interestingly, Ngaoundere and Garoua also had higher levels of liver lesions (although the numbers are small) and were also abattoirs with high fluke burdens. We also recorded the four measures of pathological changes and the comparison of the distribution of lesions, type and size is given in the PCA in Fig. 5. This suggests that the pattern and severity of lesions were different in the different abattoirs and both the first and second principal components were statistically significantly different (p < 0.01) between abattoirs based on an ANOVA. For PC1 pairwise comparisons showed Bamenda different from the northern abattoirs which appear to have larger and more extensive lesions and for PC2 all pairwise comparisons were significant except between Garoua and Maroua.

The lesion data were collapsed to the animal-level and the summary results are presented in Table 2. Although the sample sizes are much smaller for Garoua and Maroua, the raw estimates suggest that the prevalence of animals with bTB-like lesions is significantly lower in Bamenda compared to the other 3 abattoirs in the northern Regions with Garoua having the highest prevalence, ∼6 times that of Bamenda.

### Distribution and prevalence of mycobacterial species isolated from cattle from slaughter cattle from the 4 municipal abattoirs in cameroon

The majority of animals with bTB-like lesions had *M. bovis* isolated from at least one cultured lymph node (150/201) in comparison to only 3/179 randomly selected animals with no bTB-like lesions where a single retropharyngeal lymph node was cultured. Based on culture as a gold standard the sensitivity of lesions as a diagnostic test for *M. bovis* at the animal level was 86% (81–90%) and the specificity was 80% (74–85%). Further work is being done using no gold standard approaches to estimate these more reliably. This equates to a positive predictive value of 75% (61–86%) in Bamenda and 78 (69–85%) in Ngaoundere. In addition, a number of other mycobacteria were also isolated from these animals (Table 2) including one animal with *M. tuberculosis* and 35 with non-tuberculous mycobacteria (NTMs) and 4 animals with mixed *M. bovis* and NTM infections.

**Figure 6:**
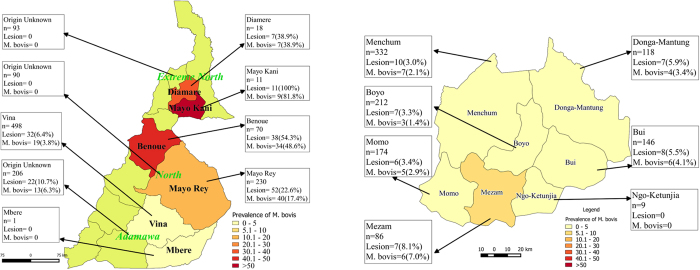
(**a**) Chloropleth map showing the minimum estimated mean prevalence of *M. bovis* by reported administrative Division of origin in the North West Region for animals slaughtered in Bamenda abattoir, Cameroon and (**b**) Chloropleth map showing the estimated mean prevalence of *M. bovis* by reported administrative Division of origin in the three Northern Regions for animals slaughtered in Ngaoundere, Garoua and Maroua abattoirs, Cameroon. (Fig. 6a,b were *generated using QGIS 2.2^®^ and shp files obtained from the GADM database of Global Administrative Areas (*www.gadm.org)).

The spatial distribution of *M. bovis* positive cattle is plotted in Fig. 6 based on a subset of n = 2088 animals where the butcher or owner’s view of the origin of the animal was reported. There were 52 missing origins in the Northwest Region and 206 for the 3 northern Regions. In the Northwest Region apart from the Mezam Division, which had the highest prevalence of *M. bovis* of 6.9%, all the other Divisions that supply Bamenda municipal abattoir had a prevalence of less than 5% (Fig. 6a). By comparison most of the cattle slaughtered in the Extreme North and North Regions of Cameroon came from Mayo-Kani and Benoue Divisions with extremely high apparent prevalences of *M. bovis* of 81.8% (9/11) and 48.6% (34/70) as shown in Fig. 6b. Conversely, cattle from the Vina Division in the Adamawa Region had the lowest burden 3.8% (19/498) of all the Divisions supplying the 3 northern abattoirs. The patterns are very similar if bTB-like lesions prevalence distribution is plotted instead of *M. bovis* prevalence (not shown). These estimates however need to be interpreted with some caution as the butchers often did not know the origins of the animals and so the maps are based on small and potentially biased subsets of the data.

The *M. tuberculosis* isolate came from a non-lesioned retropharyngeal lymph node from a bull, with a dentition score 2 and body condition score of 3 and it originated from the Momo Division. The unadjusted prevalence of *M. tuberculosis* in slaughter cattle in Bamenda was therefore an estimated 0.09% (0.00–0.49%).

The NTMs were broken down into the different species and whether they came from a bTB-like lesion or a “normal” lymph node in Table 3. Five different NTM species were identified using the Hain Genotype CM or AS kits; *M. fortuitum*, *M. gordonae*, *M. mucogenicum*, *M. phlei and M. scrofulaceum*. Of the 35 isolates screened with the Hain Genotype CM or AS kits, 32 were identified as NTM with the species of 18 out of the 32 deduced while the species of 14 could not be deduced. The 14 are probably NTMs not commonly encountered in human mycobacteriosis since the kits are optimized for detecting only 37 common NTMs. There were also three Gram-positive bacilli that did not belong to the genus *Mycobacterium*, these were recovered in samples in Bamenda and Ngaoundere municipal abattoirs but were not typed further. *M. fortuitum* was recovered from animals with visible lesions only at all abattoirs except Maroua. *M. gordonae* on the other hand was only recovered from animals that presented with bTB-like lesions at Bamenda municipal abattoir. *M. phlei,* which was the most dominant NTM species, was recovered from both animals that presented with and without bTB-like lesions at slaughter at the Bamenda and Ngaoundere municipal abattoirs (Table 3).

If this pattern of NTMs were repeated across all non-lesioned animals, the estimated prevalence in Bamenda (17/91) would be 18.7% (12.4–30.8%) and in Ngaoundere (4/88) would be 4.5% (1.1–9.6%), from a single retropharyngeal LN from a random sample of non-lesioned animals. For animals with bTB-like lesions the prevalences of NTMs would be 6.7% (1.4–18.3%) for Bamenda and 6.6% (2.7–13.1%) for Ngaoundere. Although the odds were 1.67 (0.83–3.70) times higher for recovery of an NTM from a non-lesioned animal than form one with bTB-like lesions this was not significant at the 95% level.

The 4/2,346 animals that had mixed infections represents a possible prevalence of ∼0.17% (0.05–0.44) overall. All the four co-infections were with *M. bovis* and an NTM. Two of these were with the unknown mycobacteria species cultured from Garoua and Maroua municipal abattoirs. The other 2 were co-infections with *M. phlei* and *M. fortuitum* in cattle slaughtered at Ngaoundere and Garoua municipal abattoirs respectively (Table 2).

### Risk factor analysis for bTB-like lesions in cattle at slaughter in cameroon

The univariable analysis (Table 4) shows a large and significant increased odds of occurrence of bTB-like lesions in the northern abattoirs compared to Bamenda as already discussed but quantified here. One of the key questions was whether this difference was real or was due to the observed differences in structure of the populations in the different abattoirs. Interestingly there was also an increased odds of bTB-like lesions in thin animals, older animals and female cattle. A multivariable model was developed (Table 5) and all the variables from the univariable analysis were included in the final model. Interactions and confounding between age and sex were checked but there was no evidence of either although in the final model neither are statistically significant at the 5% level their inclusion improves the AIC so they were retained. Further the adjusted prevalence estimates based on a standardised population structure (in this case the structure of the Bamenda population of slaughter cattle) were 4.0% (2.8–5.1%) for Bamenda, 15.1% (10.3–21.9) for Ngaoundere, 17.5% (11.1–26.6%) for Garoua and 12.2% (6.8–20.1) for Maroua. This provides strong evidence that after adjusting for the various potential confounders and the population age structure the northern abattoirs had significantly higher bTB-like lesion prevalences and by implication much higher levels of *M. bovis*.

### Risk factor analysis for *M. bovis* in cattle at slaughter in cameroon

A univariable analysis for the isolation of *M. bovis* from a sampled tissue from an animal was carried out using the subset of animals from which at least one lymph node or tissue was collected and cultured (n = 323) (Table 6). Animals with multiple tissues cultured were classified as confirmed positive for *M. bovis* if one or more cultures from that animal were typed as *M. bovis* using the Hain kit. The final dataset consisted of 144 animals with bTB-like lesions for which complete data was available for all variables of interest and 179 without bTB-like lesions. A multivariable logistic regression model was then developed and the final model included abattoir, sex and breed (Table 7). Ngaoundere had 2.6 times the odds of being proven *M. bovis* positive compared to Bamenda and Gudali/mixed breed were at considerably lower odds of being positive compared to the White Fulani breed. Animal sex was retained although not statistically significant as it improved the overall fit of the model based on the AIC. If the presence of a lesion was included as a predictor all other risk factors drop out as this overwhelms other variables as it is so strongly linked to *M. bovis*.

## Discussion

The aim of this study was to get current estimates of the prevalence and identify risk factors for bTB-like lesions, describe the diversity of mycobacterial infections and estimate the minimum prevalence of *M. bovis* in slaughter cattle across Cameroon. This represents the most extensive study of mycobacterial infections in Cameroon and has provided important baseline data for understanding bovine tuberculosis and informing public health on the risks of zoonotic tuberculosis. Cameroon has a large cattle population ∼5–6 million with extensive movements of cattle within Cameroon and also from Chad and Central African Republic through Cameroon to Nigeria and these results also have useful information for public health authorities in the neighbouring countries.

We identified a number of mycobacterial species dominated by *M. bovis* from lesions found at slaughter with the exception of 3 isolates from grossly normal lymph nodes. In addition, we identified a bovid with *M. tuberculosis* as has been reported in other studies in Ethiopia, Burkina Faso, Nigeria, Zambia, India and Spain^31^,^32^,^33^,^34^,^35^,^36^. It is currently not clear whether this could be transmitted on to other cattle or spill back into the human population.

Non-tuberculous mycobacteria (NTM) were predominantly recovered from cattle with and without lesions and from Bamenda and to a lesser extent Ngaoundere and as reported elsewhere in Africa and Europe^37^,^38^,^39^. *M. phlei*, a saprophyte and rare pathogenic *Mycobacterium*^40^,^41^ was the most recovered NTM followed by *M. fortuitum* in Cameroon. This rare pathogen has been previously reported in both pulmonary and extra-pulmonary TB patients in Oregon in the USA and Asia although in lower proportions^42^,^43^. The diversity and species profile in Cameroon is slightly different from that reported in Chad^44^, but some commonly recovered species like *M. gordonae* were recovered. The former and *M. fortuitum* are so ubiquitous that they have previously been recovered from human, animals and the environment elsewhere^45^,^46^,^47^,^48^. Interestingly, a study in Nigeria found an association (OR = 2.54) with NTMs in human and seasonal Harmattan winds^49^. The vast majority of the NTMs in this study were recovered from the retropharyngeal lymph nodes consistent with oral exposure from the environment^50^,^51^,^52^.

The prevalence of bTB-like lesions after adjusting for the different population structure of slaughter cattle in each abattoir varied significantly between abattoirs with the lowest prevalence in Bamenda of ∼4% in the Northwest Region compared to 12–17% in the three northern Region abattoirs. Previous studies in the Northwest Region had shown a considerably lower prevalence of bTB-like lesions in the Bamenda abattoir^12^ although a subsequent study suggested an increasing trend in prevalence from about 2003 to 2010 which if projected to 2012 seems consistent with our estimate^13^. It is not at all clear why there seems to have been such a dramatic increase in the last few years and this potential changing situation may have important implications particularly for the developing dairy industry in the region^53^. At the National level, the overall bTB-like lesion estimate is slightly higher than the 7.3%, 6.7% and 6.4% reported in Chad, Burkina Faso and Nigeria respectively^54^,^55^,^56^. It should be noted that cattle are known to be transported from Maroua for slaughter in N’djamena, Chad^15^, therefore the Extreme North Region of Cameroon is likely to be contributing to the lesion estimates documented in Chad. This study was a cross-sectional study and so temporal and seasonal variation, are not captured here.

The prevalence estimates for *M. bovis* were all slightly lower than those for bTB-like lesions but the overall pattern was not changed across the 4 abattoirs. *Mycobacterium* are notoriously difficult to culture so it is not surprizing that not all lesions produced a positive culture, however, given the very strong association between bTB-like lesions and a positive *M. bovis* culture it is likely that most of these were *M. bovis*.

Using data from the butchers on the market of origin of their animals, although incomplete particularly for the northern abattoirs, we were able to identify more precisely administrative division of the study area with particularly high prevalence such as Mezam in the Northwest Region and the Divisions of Mayo Rey and Benoue in the Northern Region and Mayo Kani and Diamare in the Extreme North Region. There is potentially much more mixing of cattle in the northern regions where animals are known to cross from the Central African Republic and Chad (from Sudan) and many continue on into Nigeria where there are even larger markets for meat than in Cameroon^57^. These areas are relatively isolated from the higher plateau areas of the Adamawa and mountainous areas of the Northwest and this information could help develop policies to limit the spread of bTB form these higher prevalence areas. As already mention, however, there may be biases both in the sampling as we were only able to sample for short periods in each abattoir and for Maroua and Garoua these were even shorter. We may have sampled on a particularly good or bad week and are unable to know what biases we have and so further studies in these areas is needed to build a more complete picture. Also the butchers were reporting what they believed to be the origins of the animals but may reflect the location of the last market the animal came from rather than it real origin.

The risk of having bTB-like lesions was associated with age and body condition as might be expected and reported in previous studies in Africa^58^,^59^. Interestingly there was a strong association with breed and risk of having a bTB-like lesion with Holstein-Frisians at high risk; ∼11 times higher odds compared to the white Fulani breed. Although the number of Holstein-Frisians in the study was small, this is consistent with other studies in Ethiopia^23^ and in Cameroon (although the association was not statistically significant based on the authors design)^60^. Further the Gudali breed seems to be protected against bTB-like lesions when compared to White Fulani, which was supported by anecdotal reports from the butchers. Further the model also suggests there is some evidence of animals with lesions having increased odds of being in poor condition consistent with potential clinical impact of disease. Other factors which we were not able to record for these cattle but reported as risk factors are transhumance^61^ and wildlife^62^. Importantly therefore even after adjusting for other variables and confounders there is a significantly higher prevalence of bTB-like lesions in slaughter cattle in the more northern abattoirs. This may reflect different stages of the ongoing epidemic of bTB across Africa and is important to understand particularly in the light of the growing dairy sector in Cameroon^53^ and many other SSA countries.

The distribution of bTB-like lesions within animals showed that lesions were mostly affecting the retropharyngeal, mediastinal and bronchial lymph nodes reflecting most likely routes of infection through the respiratory route or ingestion although the dominance of site varied between abattoirs with more retropharyngeal involvement in animals slaughtered in Bamenda and Ngaoundere compared to more mediastinal involvement in those in Maroua in the Extreme North Region. We have no data to help unravel why this difference might exist but it may relate to route of exposure and factors such as feeding supplements, such as salt or cotton seed cake, which might be increasing close head to head contact and risk of ingestion. In general this finding agrees with previous reports from the Bamenda abattoir in Cameroon^12^ and elswhere^22^,^63^,^64^. Interestingly there appear to be marked differences between the abattoirs in terms of lesion distribution (although the numbers are small for Garoua and Maroua) but of particular interest is that there seem to be possibly higher levels of liver involvement in Ngaoundere compared to Bamenda and this correlates with much high fluke infection levels observed at slaughter (unpublished). At the time of sampling the meat inspection procedure in Ngaoundere did not include the inspection of the carcase head (this was done by team members for the period of the study), therefore from a public health point of view more infected animals might be missed in light of the observed lesion distribution. This issue was discussed with the butchers and government meat inspectors at a stakeholder meeting after the end of the study. Previous reports from Ethiopia found high levels of infection in the respiratory related lymph nodes in housed cattle compared to digestive tract in extensively reared cattle^23^. Given that most if not all the cattle slaughtered in this study will have be extensively grazed it is unclear why there should be such a difference in lesion distribution but may be due to different strains in the different Regions.

Bovine tuberculosis remains an important disease problem both due to its impacts on livestock production and due to its zoonotic potential. The current study has highlighted the high prevalences of bTB in sections of the cattle population and this will be having significant impacts on production and animal welfare. Subsequently negatively impacting cattle rearing communities livelihoods and potential zoonotic transmission to farmers, herdsmen and slaughterhouse workers and the traders and consumers of milk^65^. The identification of *M. tuberculosis* in cattle further highlights the extremely complex nature of the problem with bi-directional transmission between humans and cattle in these settings. Although the current human disease burden is mostly due to *M. tuberculosis*, as control efforts from WHO and others as part of the “END-TB” programme by 2035 bring down this burden the importance of *M. bovis* and NTMs will increase^66^. This may be further exacerbated by the rapid rise in dairy production across Africa. Furthermore, these high rates of bTB in livestock populations, in this setting, make control options such as test and slaughter, as used in high-income countries, impractical. Hence in sub-Saharan Africa, possibly more than any other region of the world, is likely to benefit from development of an effective *M. bovis* vaccine. The levels of zTB in the associated human populations is not well documented for these areas but general estimates of ∼3% zTB in the human TB population represents around 100,000 cases in Cameroon^67^. In the absence of any other controls these results emphasise the importance of maintaining high standards of meat inspection. Also given the relative low levels of knowledge of the risks of zoonotic tuberculosis in Cameroon but particularly in the Vina^68^, it also highlights the need for better public health engagement^68^ and raising of awareness to reduce transmission via milk as well as direct contact.

## Conclusion

Our study has shown that mycobacterial disease in cattle is widespread throughout Cameroon with *M. bovis*, *M. tuberculosis* and a number of NTMs present in cattle destined for human consumption. *M. bovis* infections dominated with much higher prevalences across the northern Regions (Ngaoundere, Garoua and Maroua) with higher cattle movements with neighbouring countries, compared to that in animals supplying Bamenda abattoir (Northwest Region). There also appears to be some breed differences in risk with the Fulani breeds at increased risk. This does however offer some potential for selection of more resistant breed lines. Finally the high prevalence of *M. bovis* and the presence of *M. tuberculosis* in cattle highlight the risks of (reverse) zoonotic transmission and the need for action if this important disease is to be controlled in humans and cattle.

## Additional Information

**How to cite this article**: Egbe, N. F. *et al.* Abattoir-based estimates of mycobacterial infections in Cameroon. *Sci. Rep.* **6**, 24320; doi: 10.1038/srep24320 (2016).

## Figures and Tables

**Table 1:** The population structure (with sample proportions) of the animals slaughtered in 4 municipal abattoirs in Cameroon in 2013.

Variable		Abattoir			
		Bamendan = 1129	Ngaounderen = 935	Garouan = 160	Marouan = 122
Sex	Male	689 (0.610)	79 (0.085)	16 (0.100)	29 (0.238)
	Female	440 (0.390)	853 (0.912)	139 (0.869)	93 (0.762)
	NA		3 (0.003)	5 (0.031)	
Breed	Exotic	5 (0.004)	0 (0.000)	0 (0.000)	0 (0.000)
	Gudali	0 (0.000)	16 (0.017)	0 (0.000)	0 (0.000)
	Mixed	170 (0.151)	626 (0.670)	16 (0.100)	24 (0.197)
	Red Fulani	429 (0.380)	101 (0.108)	40 (0.250)	45 (0.369)
	White Fulani	525 (0.465)	187 (0.200)	104 (0.650)	52 (0.426)
	NA	–	5 (0.005)	–	1 (0.008)
Dentition	0	32 (0.028)	28 (0.030)	0 (0.000)	0 (0.000)
	1	124 (0.110)	21 (0.022)	0 (0.000)	0 (0.000)
	2	206 (0.182)	81 (0.087)	1 (0.006)	0 (0.000)
	3	223 (0.198)	234 (0.250)	25 (0.156)	2 (0.016)
	4	510 (0.452)	484 (0.518)	120 (0.750)	74 (0.607)
	5	30 (0.027)	75 (0.080)	14 (0.088)	46 (0.377)
	NA	4 (0.004)	12 (0.013)	–	–

**Table 2:** Animal-level summary table of lesions detected and Mycobacterial species recovered by culture from slaughtered cattle in Cameroon.

Abattoir (Region)	No.animalsinspected	No. lesioned/non-lesionedanimals cultured	% Lesioned (95% CI)*	Culture andZN positive	No. lesioned/non-lesionedanimals with*M. bovis*	Min. % animalswithM. bovis*	*M. tuberculosis*	*M. bovis*/NTM	NTM	Gram-positivebacilli
Bamenda (Northwest)	1129	45/91	3.99 (2.92–5.30)	53	31/0	2.75 (1.87–3.87)	1	0	20	1
Ngaoundere (Adamawa)	935	106^a^/88	11.33 (9.37–13.5)	85	69/3	7.70 (6.07–9.60)		1	11	2
Garoua (North)	160	38/0	23.75 (17.39–31.11)	35	34/NA	21.3 (15.19–28.41)		2	3	
Maroua (Extreme North)	122	18/0	14.75 (8.98–22.30)	16	16/NA	13.1 (7.69–20.42)		1	1	
**All abattoirs**+	**2346**	**207^a^/179**		**189**	**150/3**		**1**	**4**	**35**	**3**

^*^Exact binomial confidence intervals.

^+^Overall prevalence estimates not given as weightings needed to adjust for sampling are unknown.

^a^N.B. 6 animals with lesions in Ngaoundere abattoir were not cultured.

**Table 3:** Summary table of non-tuberculous mycobacteria species recovered from slaughtered cattle in 4 abattoirs in Cameroon stratified by whether they were recovered from an animal reported as having bTB-like lesions by the meat inspector or from the random sample of lymph nodes from non-lesioned animals.

Species	Abattoir				
	Bamenda	Ngaoundere	Garoua	Maroua	Total
Lesioned animals	n = 45	n = 106	n = 38	n = 18	
*M. fortuitum*	1	3	1	0	5
*M. gordonae*	0	0	0	0	0
*M. mucogenicum*	0	1	0	0	1
*M. phlei*	1	1	0	0	2
*M. scrofulaceum*	0	0	1	0	1
*M. species*	1	2	1	1	5
Total from lesioned animals	**3 (0.067)**	**7 (0.066)**	**3 (0.079)**	**1 (0.056)**	**14**
Non-lesion animals	**n = 91**	**n = 88**			
*M. fortuitum*	0	0	–	–	0
*M. gordonae*	2	0	–	–	2
*M. mucogenicum*	0	0	–	–	0
*M. phlei*	8	1	–	–	9
*M. scrofulaceum*	1	0	–	–	1
*M. species*	6	3	–	–	9
Total from non-lesioned animals	**17 (0.187)**	**4 (0.045)**	**–**	**–**	**21**

Number in bold parentheses are proportions.

**Table 4:** Univariable analysis of factors associated with the presence of a bTB-like lesion in an animal at four municipal abattoirs in Cameroon (n = 2,346).

	Variables	No. Lesion‘+ve’	No. Lesion‘−ve’	Oddsratio	95% ConfidenceInterval	Fisher exacttest p-value
Abattoir	Bamenda	45	1084	1		<0.001
	Ngaoundere	106	829	3.08	2.13–4.52	
	Maroua	18	104	4.17	2.18–7.65	
	Garoua	38	122	7.50	4.54–12.31	
Body Condition	Poor	77	528	3.09	1.59–6.56	<0.001
	Normal	118	1371	1.82	0.96–3.81	
	Good	11	233	1		
Age (Dentition)	Juvenile	20	473	1		<0.001
	Adult	186	1651	2.66	1.65–4.51	
Sex	Male	34	779	1		<0.001
	Female	168	1357	2.84	1.93–4.27	
Breed	White Fulani	83	785	1		0.086
	Mixed/Gudali	61	791	0.73	0.51–1.04	
	Exotic	1	4	2.36	0.05–24.23	
	Red Fulani	62	553	1.06	0.74–1.52	

**Table 5:** Multivariable logistic regression model for the presence of a bTB-like lesion in an animal at four municipal abattoirs in Cameroon (n = 2,318).

	Variables	Odds ratio	95% ConfidenceInterval	Likelihood ratiotest
Abattoir	Bamenda	1		<0.001
	Ngaoundere	4.29	2.17–6.74	
	Maroua	3.34	1.78–6.06	
	Garoua	5.12	3.00–8.72	
Body Condition	Good	1		0.006
	Normal	1.56	0.84–317	
	Poor	2.41	1.26–5.02	
Age (Dentition)	Juvenile	1		0.067
	Adult	1.57	0.94–2.59	
Sex	Male	1		0.168
	Female	1.36	0.87–2.10	
Breed	White Fulani	1		0.001
	Mixed/Gudali	0.49	0.33–0.74	
	Exotic	11.37	1.18–109.97	
	Red Fulani	1.31	0.90–1.91	

**Table 6:** Univariable logistic model for confirmed positive *M. bovis* in an animal at two municipal abattoirs in Cameroon (n = 323).

Variables	Levels	No. *M.bovis* valign="middle"‘^+^ ve’	No. *M.bovis* valign="middle"‘^−^ve’	Odds valign="middle"ratio	95% Confidence valign="middle"Interval	Fisher exact valign="middle"test p-value
Abattoir	Bamenda	30	105	1		0.002
	Ngaoundere	72	116	2.17	1.28–3.72	
Sex	M	18	73	1		0.005
	F	84	148	2.3	1.25–4.37	
Dentition	Juvenile	14	53	1		0.039
	Adult	87	167	1.97	1.01–4.06	
Condition	Normal	57	144	1		0.105
	Poor	38	56	1.71	0.99–2.95	
	Good	7	19	0.93	0.31–2.47	
Breed	White Fulani	38	68	1		0.357
	Red Fulani	29	60	0.87	0.46–1.63	
	Gudali/Mixed	35	93	0.67	0.37–1.22	

Exotic breed left out of analysis because only a single animal was included in this subset.

**Table 7:** Multivariable logistic regression model for confirmed presence of *M. bovis* in an animal at two municipal abattoirs in Cameroon (n = 321).

Variables	Levels	Oddsratio	95% ConfidenceInterval	Likelihoodratio testp-value
Abattoir	Bamenda	1		0.003
	Ngaoundere	2.63	1.38–5.11	
Sex	Male	1		0.123
	Female	1.71	0.86–3.40	
Breed	White Fulani	1		0.005
	Red Fulani	1.02	0.54–1.91	
	Gudali/Mixed	0.40	0.21–0.75	
